# Facial Mechanosensory Influence on Forelimb Movement in Newborn Opossums, *Monodelphis domestica*

**DOI:** 10.1371/journal.pone.0148352

**Published:** 2016-02-05

**Authors:** Marie-Josée Desmarais, France Beauregard, Thérèse Cabana, Jean-François Pflieger

**Affiliations:** Département de sciences biologiques, Université de Montréal, Montréal, Québec, Canada; Texas A&M University, UNITED STATES

## Abstract

The opossum, *Monodelphis domestica*, is born very immature but crawls, unaided, with its forelimbs (FL) from the mother's birth canal to a nipple where it attaches to pursue its development. What sensory cues guide the newborn to the nipple and trigger its attachment to it? Previous experiments showed that low intensity electrical stimulation of the trigeminal ganglion induces FL movement in *in vitro* preparations and that trigeminal innervation of the facial skin is well developed in the newborn. The skin does not contain Vater-Pacini or Meissner touch corpuscles at this age, but it contains cells which appear to be Merkel cells (MC). We sought to determine if touch perceived by MC could exert an influence on FL movements. Application of the fluorescent dye AM1-43, which labels sensory cells such as MC, revealed the presence of a large number of labeled cells in the facial epidermis, especially in the snout skin, in newborn opossums. Moreover, calibrated pressure applied to the snout induced bilateral and simultaneous electromyographic responses of the triceps muscle in *in vitro* preparations of the neuraxis and FL from newborn. These responses increase with stimulation intensity and tend to decrease over time. Removing the facial skin nearly abolished these responses. Metabotropic glutamate 1 receptors being involved in MC neurotransmission, an antagonist of these receptors was applied to the bath, which decreased the EMG responses in a reversible manner. Likewise, bath application of the purinergic type 2 receptors, used by AM1-43 to penetrate sensory cells, also decreased the triceps EMG responses. The combined results support a strong influence of facial mechanosensation on FL movement in newborn opossums, and suggest that this influence could be exerted via MC.

## Introduction

Like other marsupials, the gray short-tailed opossum (*Monodelphis domestica*) is born in a very immature state but nonetheless crawls, unaided by the mother, from the urogenital opening to a nipple where it attaches to pursue its maturation [1–3]. To do so, the newborn opossum performs rhythmic and alternate movements of the forelimbs (FL), while its trunk sways from side to side, but its hindlimbs are immobile [2]. In mammals limb movements depend on neuronal networks located in the spinal cord, which are modulated by sensory afferents and by projections from the brain [4–6]. Sensory clues are certainly needed to guide the newborn opossum to a nipple and trigger its attachment to it. On the basis of anatomical observations, mechanosensation from the face has been postulated as a guide for some other marsupial species [7–9]. When crawling towards the teat, the snout of the newborn opossum is in regular contact with the mother's belly, potentially stimulating sensory afferents associated with the trigeminal nerve that are present in the facial skin at that age. Indeed, the newborn reacts to gentle pressure manually exerted on the snout by extending its FL.

In previous experiments [10], we compared the effect of electrical stimulations of the trigeminal ganglion, the olfactory bulbs, the superior colliculus and the vestibular complex on FL movement of newborn opossums in *in vitro* preparations. We found that the trigeminal ganglion required the lowest stimulus intensity to elicit FL extensions resembling those performed by intact animals. Moreover, using immunohistochemistry to detect NF-200, a marker of nerve fiber maturation, we revealed the presence of trigeminal fibers into the snout skin. However, Nissl stained histological sections showed no large encapsulated receptors such as Vater-Pacini and Meissner corpuscles, and the developing hair follicles are still internal in the snout of the newborn opossum [10]. Using electron microscopy, Jones and Munger [11] documented the presence of numerous Merkel cells (MC) in the snout epidermis of newborn opossums, some of which seemed mature and connected to nerve terminals. MC are epidermal mechanoreceptors which convey light touch and are contacted by slowly adapting type I (SA I) fibers (reviews in [12–15]). Like other sensory receptors, MC use glutamate as neurotransmitter [13,15]. In the head, MC are innervated by fibers from the trigeminal ganglion.

Herein, we report on a series of experiments carried out to further substantiate the possible role of mechanosensory inputs conveyed by the trigeminal nerve on motor guidance of newborn opossums, and more specifically touch mediated by Merkel cells. Firstly we have used AM1-43 labeling *in vivo* to reveal the presence and distribution of putative MC in the epidermis of newborn opossums. AM1-43 is the fixable analog of FM1-43, and both styryl pyridinium dyes have been used to label a range of sensory cell types *in vivo*, including MC in the epidermis of mammals [16–18]. This dye is thought to enter cells at least via purinergic type 2 receptors [17,19]. Secondly, we have recorded the electromyographic (EMG) responses of the triceps muscle of both FL following application of calibrated forces on the snout skin in *in vitro* preparations of newborn opossums. Thirdly, we have applied an antagonist of the metabotropic glutamate 1 receptors (YM298198) involved in MC transmission [20] to verify if the disruption of MC activity affects FL EMG responses. Moreover, we have applied an antagonist of the purinergic type 2 receptors, the receptors used by AM1-43 to enter sensory cells, to verify its effect on FL EMG responses. The results obtained support the hypothesis that touch sensitivity of the snout relayed by the trigeminal system is functional in newborn opossums and may influence its FL movements, possibly contributing to guiding the animal to the nipple or its attachment to it, and that this action could be mediated by Merkel cells.

## Materials & Methods

This research was performed under the guidelines of the Canadian Council on Animal Care and the present study was approved by the University Comité de déontologie de l’expérimentation sur les animaux (CDEA). Gray, short-tailed opossums *Monodelphis domestica* were obtained from a colony maintained at the departmental animal facility. Opossums being solitary animals, they are maintained in individual cages (about 0.1 m^2^ of foot surface) containing an opaque PVC half-pipe for shelter and a plastic round nest as used for laboratory rodents. They are provided water and fox chow pellets (Moulées A&M Mathieu Inc., Quebec, Canada) ad lib complemented weekly with fresh vegetables and fruit. Female oestrus is induced by the presence of a male. For breeding, a male and a female are placed in a larger cage separated in two halves by a perforated grid for 7 days before removing the grid. The animals are then left in contact for 4 days before being transferred to their original cages. As gestation lasts 14–15 days, the female is checked daily for the presence of newborns from the 10^th^ to the 15^th^ day after the grid was removed from the breeding cage. Animal health is monitored regularly by animal facility personals, and procedures in case of injury have been established with the help of university veterinarians. More details about opossum husbandry and care can be found in [1,3,21,22].

Hypothermy is used to anesthetize newborn opossums because they do not regulate their temperature and become unresponsive to stimulation fast. It also minimizes the risks of over-anesthetizing neonatal animals. At the ages considered in this study, the cerebral cortex of opossums is very immature and the ascending pathways are only beginning to form, making pain consciousness unlikely [23]. Nevertheless *in vivo* experiments are kept as short as possible, and are replaced by experiments on *in vitro* decerebrated preparations, when doable.

### AM1-43 labeling

22 opossums, measuring around 1 cm long and weighting about 100 mg, were removed from their mother on the day of birth (Postnatal day 0, P0). They were lightly anesthetized by hypothermia (1–2 min at -20°C in a gauze-covered petri dish) before being injected i.p. with 0.03 ml of phosphate buffer saline (0.1 M PBS, 0.9% NaCl) alone in 5 control specimens or with AM1-43 (Biotium, USA) (3 mg/kg body weight) in 17 experimental specimens. All were put on moistened gauze in a petri dish under a lamp (30°C) for one hour and were constantly monitored. They were then deeply anesthetized by hypothermia (4–5 min at -20°C) until unresponsive to pinching of the tail or limbs, and rapidly decapitated and eviscerated. The head and the body with the FL attached were fixed by immersion in 4% paraformaldehyde (in 0.1 M PBS) for 48 h before being transferred to a sucrose solution (30% in PBS) for 24 h. Each specimen was embedded in Tissue-Tek OCT Compound (Miles Scientific) solidified in a 2-methylbutane bath frozen in carbon ice-ethanol and cut at 20 μm on a cryostat (CM3050S Leica). The sections were mounted onto Superfrost slides (Fisher) and allowed to dry for 24 h before being coverslipped using Fluoromount-G (Southern Biotech, USA).

The sections were observed with a BX51 Olympus microscope equipped for epifluorescense using filters to detect AM1-43 (excitation 450–490 nm, emission 560–620 nm) (Chroma Technology Corp) and Ultra Violet (excitation 358 nm; emission 461 nm) to verify the specificity of the labeling. Microphotographs were acquired with a color digital camera (QImaging) and digitized using the image analysis software Image-Pro Plus 7.0 (Media Cybernetics). Microphotographs were also taken using a laser scanning confocal microscope (TCS SP2, Leica Microsystems) with 40X (NA:1.25) or 100X (NA:1.40) 488 nm and 633 nm lasers. Conversion to monochrome format and manual adjustment of contrast were made in Corel Photo-Paint12. Figures and drawings were made using CorelDraw12. Counts of AM1-43 labeled cells were made from serial sections in one specimen.

### FL EMG responses to mechanical stimulation of the snout and pharmacological manipulation

A total of 27 opossums aged P0 to P4 were used for the electrophysiological experiments reported here. Opossums up to P4 were used to ensure a sufficient number of animals. The neuraxis with the limbs attached were prepared as described previously for *in vitro* experiments [10,24]. Briefly, each opossum was deeply anaesthetized by hypothermia and placed in a Sylgard-lined petri dish filled with physiological solution (NaCl 125 mM, KCl 3 mM, NaHCO_3_ 25 mM, NaH_2_PO_4_ 1 mM, MgCl_2_ 1 mM, CaCl_2_ 2 mM, Glucose 15 mM; 95% O_2_ / 5% CO_2_; pH 7.4; adapted from [25,26]). It was eviscerated and the lower jaw was removed. The skin of the body and the FL was sectioned, but the skin elsewhere was left intact. The dorsal surface of the brain and the spinal cord was exposed and the cerebral hemispheres were sectioned from the rest of the neuraxis, which was left in the carcass with the FL attached. Each specimen was then pinned to the petri dish coating, dorsal side up. Oxygenated physiological solution was perfused continually into the dish using a peristaltic pump (Watson-Marlow 120S/DM3). The preparation was kept undisturbed for at least 2 hours before the experiments began. All manipulations were carried out at room temperature (21–24°C).

**Fig 1:**
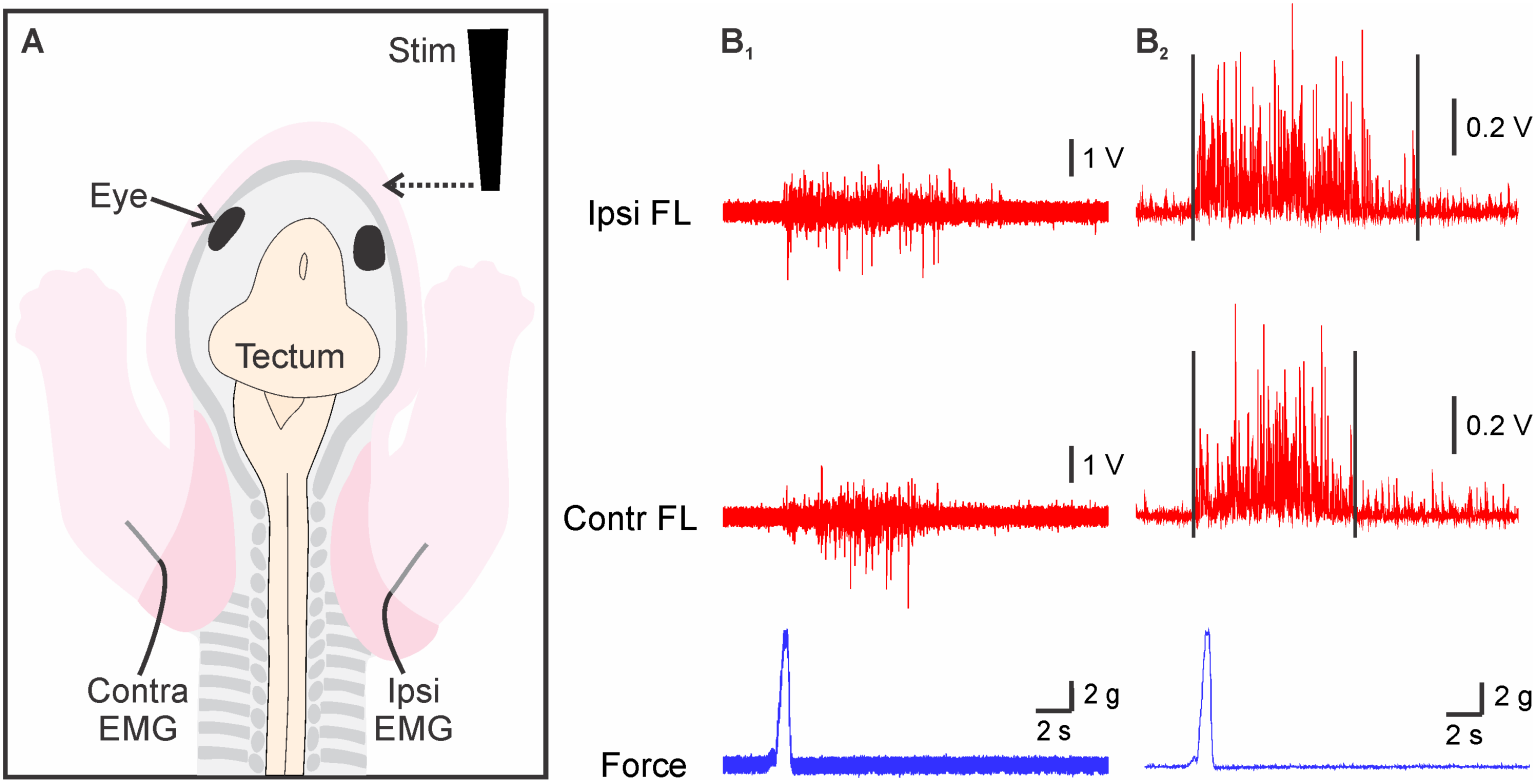
Experimental protocol for *in vitro* experiments. (**A**) Schematic representation of a preparation of a newborn opossum illustrating how forelimb (FL) responses induced by snout skin stimulation (Stim) were recorded by electromyography (EMG) of the triceps muscle from both FL. (**B**_**1**_) Raw EMG of the ipsilateral (Ipsi) FL (*top*), the contralateral (Contra) FL (*middle*) and raw force trace (*bottom*). (**B**_**2**_) EMG of the Ipsilateral FL (*top*), the Contralateral FL (*middle*) and the force (*bottom*) once rectified and with a reduced sampling rate.

To record FL movement, the triceps muscle was selected for electromyography because its contraction yields FL extensions similar to those observed in intact animals left on the mother. A teflon-insulated silver wire (wire diameter: 76.2 μm; total diameter: 139.7 μm; A-M Systems, Inc.) was inserted into the triceps of each FL as schematized in Fig 1A, and a small piece of teflon tape was tightly apposed over the muscle in order to increase stability and insulate the insertion site. The wires were connected to amplifiers (CP511, Grass Technologies) through high-impedance modules (HZP, Grass). Pressure was applied to the facial skin as described below and the amplified EMG signals (x 10 KHz; bandwidth: 3 Hz-3 KHz) were digitized (Digidata 1322A, Axon instruments), recorded (sampling rate: 11.1 KHz) and saved on disk with Clampex 9.2 software (Axon Instruments).

Mechanical pressure was applied to the facial skin using a rod probe attached to a strain gauge installed on a micromanipulator (schematized in Fig 1A; for more details see [27,28]). Pressure intensity was recorded with a Grass force displacement transducer (FT-03). The signal was amplified (× 10000) and filtered (bandwidth: 3 Hz– 3 kHz) before being digitized. Except where stated, the force used for quantitative analysis was 6–7 g. This force corresponds to 3 times the threshold at which repeatable muscular responses were induced, as determined in preliminary experiments not reported here.

The EMG signals were analyzed with Clampfit 9.2 software (Axon Instruments). Raw EMG and force signal traces (Fig 1B_1_) were rectified and the sampling rate reduced to 0.1 KHz (Fig 1B_2_). The averaged baseline trace (before the response) was adjusted at 0 V. Then, the area under the curve was measured (Fig 1B_2_) from the onset of the response, when the trace deflected upward from the baseline values, up to the return to baseline values (pre-stimulation level). In order to compare results from different limbs and different preparations, the absolute values obtained for a given limb were normalized as a percentage of the maximal area obtained for that limb. The intensity of the first 4 or 5 responses being abnormally high, presumably due to heightened excitability (see Results), they were not included in the normalization. Response latency was measured from the onset of the deflection of the gauge signal to the onset of the response (Fig 1B_2_). Statistical analyses were done using Prism 5 (Graphpad, USA). Data are presented as averages ± SEM in the text and the figures.

YM298198 (6-amino-N-cyclohexyl-N,3-dimethylthiazolo[3,2-α]benzimidazole-2-carboxamide hydrochloride, axon MEDCHEM) was applied to the bath to test the effect of disrupting MC transmission on triceps responses following snout stimulation. YM298198 is a non-competitive antagonist of the metabotropic glutamate 1 receptors which has been shown to block activity in slowly adapting type I (SA I) fibers, the fibers associated with MC, in isolated rat sinus hair follicle preparations [20]. YM298198 was dissolved at 40 μM in physiological solution before being perfused in the dish of 7 of the 27 specimens during 20 to 40 min. In 6 other specimens, we tested if purinergic type 2 receptors (P2), often associated with neuronal sensory endings (reviews in [29–31]), could be involved in the EMG responses induced by snout mechanical stimulation. This was done by applying the P2 antagonist PPADS (pyridoxalphosphate-6-azophenyl-2’,4’-disulfonic acid, Sigma-Aldrich) dissolved in physiological solution at 200 μM to the bath for 30 to 40 min. PPADS has been shown to block the entry of AM1-43, the dye used herein to identify MC, in cochlear ciliated cells [17,19]. The responses to the stimulations performed before application of either compound served as basis for comparison. Recovery of the responses following YM298198 or PPADS application was verified after rinsing with physiological solution alone.

## Results

### AM1-43 labeling

**Fig 2:**
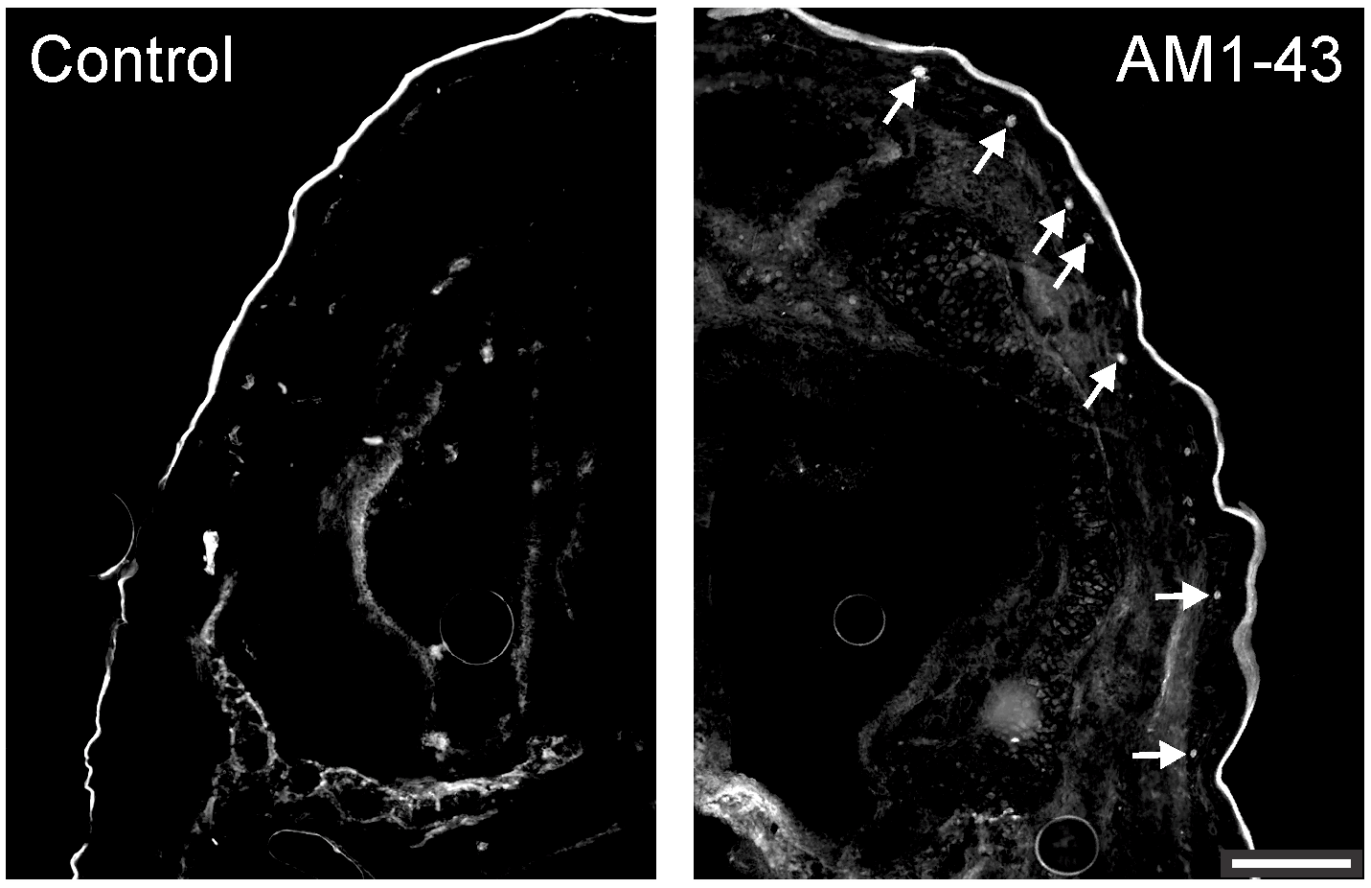
Controls for AM1-43 labeling. Transverse half-sections from the snout of one newborn opossum injected with AM1-43 (right) and one injected with the vehicle alone (left). Arrows point to labeled cells in the epidermis. Dorsal is up and medial is between photographs. The same exposure time was used and the contrast adjusted with the same parameters for both photographs. Scalebar: 100 μm.

**Fig 3:**
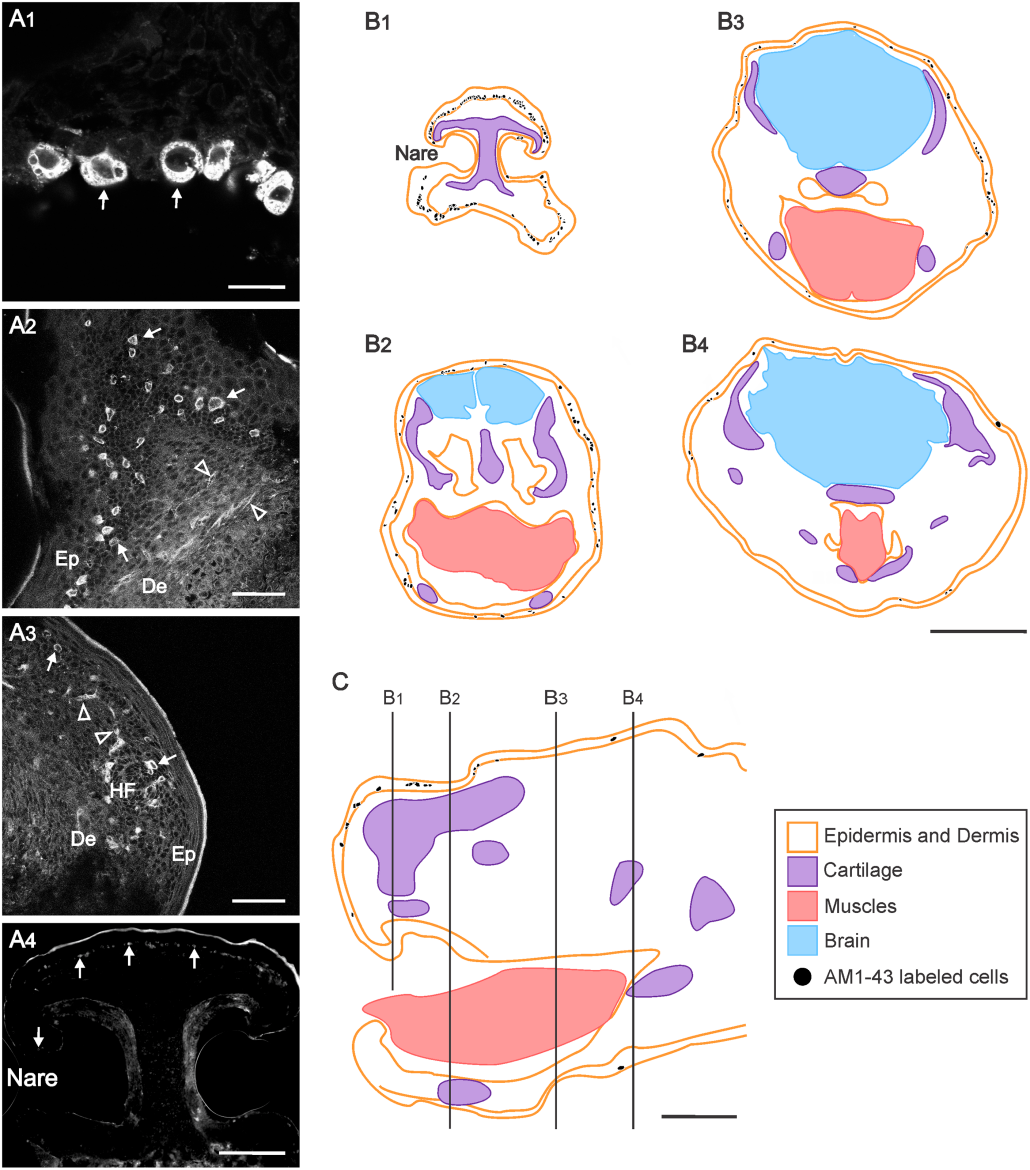
Distribution of AM1-43 labeled cells in the head of newborn opossums. (**A**) Microphotographs of head sections showing AM1-43 labeled cells (arrows) and nerve fibers (empty arrowheads) (**A**_**1**_**-A**_**4**_). Microphotographs in panels **A**_**1**_**-A**_**3**_ were obtained with a laser scanning confocal microscope (see Materials & Methods). (**B**) Schematic drawing of a sagittal section of the head of a newborn opossum indicating the levels at which the drawings in C are taken. (**C**) Cross sections from the head of another newborn opossum at the rostrocaudal levels indicated in B, illustrating the position of AM1-43 labeled cells. Legends: De, dermis; Ep, epidermis; HF, developing hair follicle. Scalebars: 20 μm in A_1_; 50 μm in A_2_ and A_3_; 200 μm in A_4_; 500 μm in B and C.

The facial epidermis of the newborn opossum consists of an epithelium of 4 to 6 layers of cells and is not yet keratinized. In AM1-43 injected specimens, fluorescent labeling was observed in facial epidermal cells which was not seen in specimens injected with the vehicle alone (arrows in Fig 2). The labeling was found in the cytoplasm, not in the nucleus (Fig 3A_1_), of cells located at the base of the epidermis of the face and in the peg of developing hair follicles (Fig 3A_3_). These labeled cells were easy to distinguish from the erythrocytes and chondrocytes located deep to the epidermis and which showed intrinsic fluorescence, the latter also visible in control specimens. In AM1-43 injected opossums, labeled nerve fibers were occasionally observed close to labeled epidermal cells (Fig 3A_2_).

Labeled cells were most abundant in the snout region, especially around the nares (Fig 3A_4_ and 3B_1_), and less abundant elsewhere on the face. They also occurred around the head and in the neck, some groupings resembling presumptive touch domes. Their distribution is plotted on drawings of transverse head sections from a representative specimen in Fig 3B and of sagittal sections from a different specimen in Fig 3C. To gain an estimate of their relative density, labeled cells were counted on every 20 sections over a length of 3160 μm from the specimen illustrated in Fig 3B. For the 1044 labeled cells counted in total on the 16 sections, they were most numerous on sections of the snout, with about 160 labeled cells per rostralmost section (Fig 3B_1_), whereas only 8 to 15 labeled cells appeared on the caudalmost head sections (Fig 3B_4_).

Occasional labeled cells were found in the buccal and nasal mucosae, as well as in the tongue epithelium, possibly in presumptive taste buds, but they were difficult to discern. No labeled cells were observed in the cephalic ganglia and in the brain, or in the epidermis of the lower neck and back. However, labeling was seen in epidermal cells of the hands and fingers, with fewer than 10 labeled cells per section.

### FL responses to mechanical stimulation of the snout

**Fig 4:**
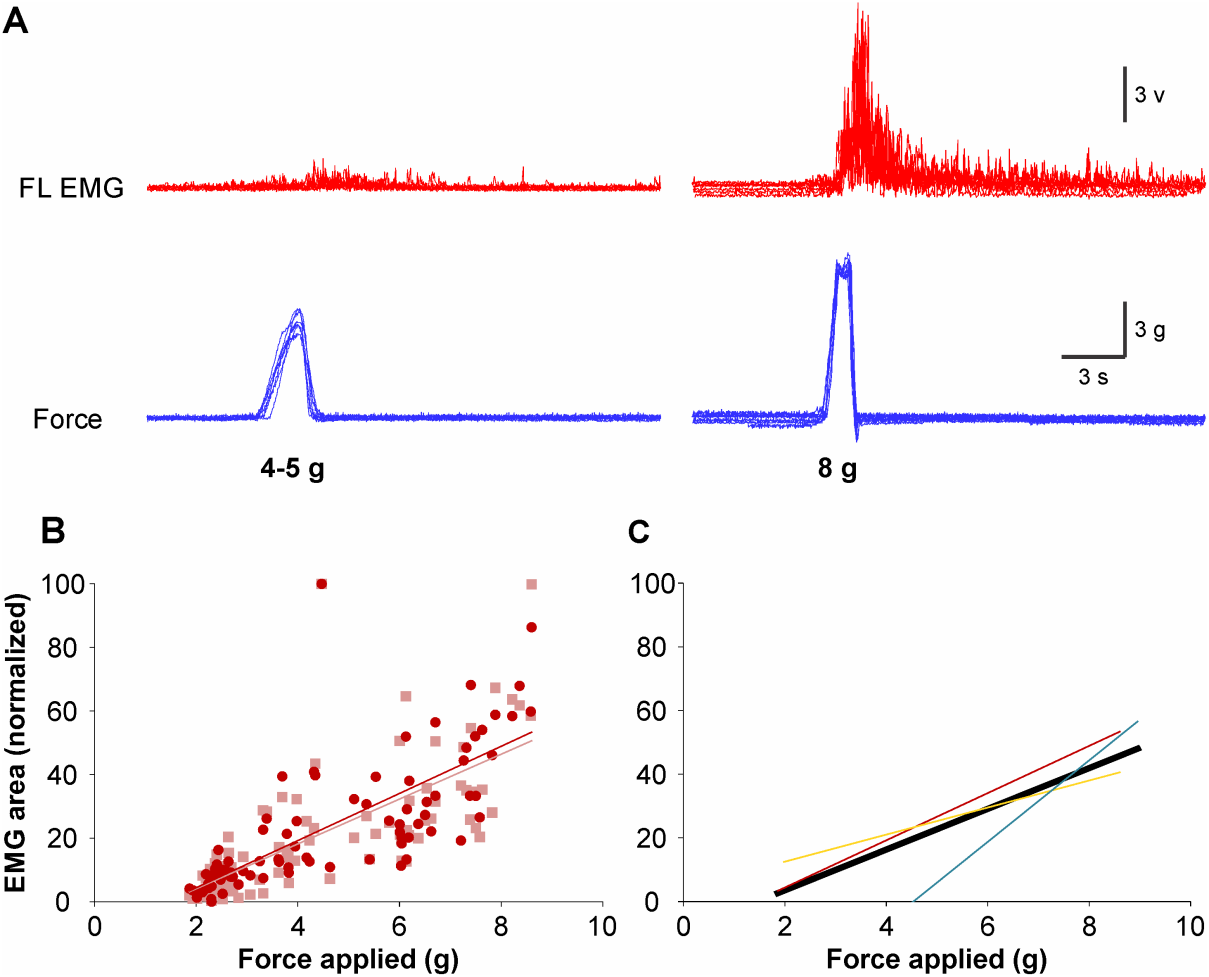
Arm extensor responses in relation to force stimulation. (**A**) EMG responses (red) of the triceps to pressures (blue) of 4–5 g or 8 g applied on the snout in one specimen. In this example, seven EMG and force traces are superimposed. (**B**) Ipsilateral (dark) and contralateral (pale) triceps responses (% EMG area, normalized) according to the strength of the force (g) applied to the snout of one specimen. (**C**) Correlation analysis for the 3 specimens tested in this series of experiment. Each color represents the regression line based on data recorded for each given specimen and the black line represents the regression line for all 3 specimens.

As mentioned above, FL movements can be elicited by tactile stimulation of the snout in intact newborn opossums. In the present *in vitro* preparations, calibrated mechanical pressure exerted on one side of the face elicited contractions of the left and right triceps, as shown by their EMG recording (Fig 4A). In the majority of cases, the response seemed to occur simultaneously on the two sides. We quantified this observation in the six specimens which served as control for the pharmacological experiments described below. The average response latencies of 136.2 ± 15.9 ms for the triceps ipsilateral to the facial stimulation and 100.2 ± 16.0 ms for the contralateral triceps (*n* = 137 stimulations) are not significantly different (P = 0.2327, Wilcoxon matched-pairs rank t-test). The amplitudes of the first 4 or 5 responses at the beginning of each recording session were generally abnormally high and may be due to hyperexcitability. Therefore, these early responses were not included in the normalization that was done to express intensity. The average response intensities in the 6 specimens were on average 27.9 ± 2.2% for the ipsilateral triceps and 21.6 ± 2.0% for the contralateral triceps (*n* = 137 stimulations). When analyzed as matched values, that is, when the ipsilateral and the contralateral responses to a given stimulation are compared, these values are significantly different (P < 0.0001, Wilcoxon matched-pairs rank t-test). This suggests that, following a given stimulation, the intensity of the response recorded on one side differs from that recorded on the other side. However, when all results for the ipsilateral triceps EMG are pooled and compared to the pooled results for the contralateral triceps EMG, the difference between left and right FL is not statistically different (P = 0.1479, Mann-Whitney t-test), indicating that, on average, response intensities recorded ipsi- and contralaterally are similar over multiple stimulations.

In three specimens we tested the effect of gradually increasing the force applied to the face, from 2 g to 9 g. We observed a proportional increase in EMG intensity (Fig 4A) which was similar in the ipsilateral and contralateral triceps (dark-red and light-red regression lines, respectively, in Fig 4B). The correlation is statistically significant (r = 0.6642; P < 0.0001, Spearman non-parametric two-tail) (black line in Fig 4C).

**Fig 5:**
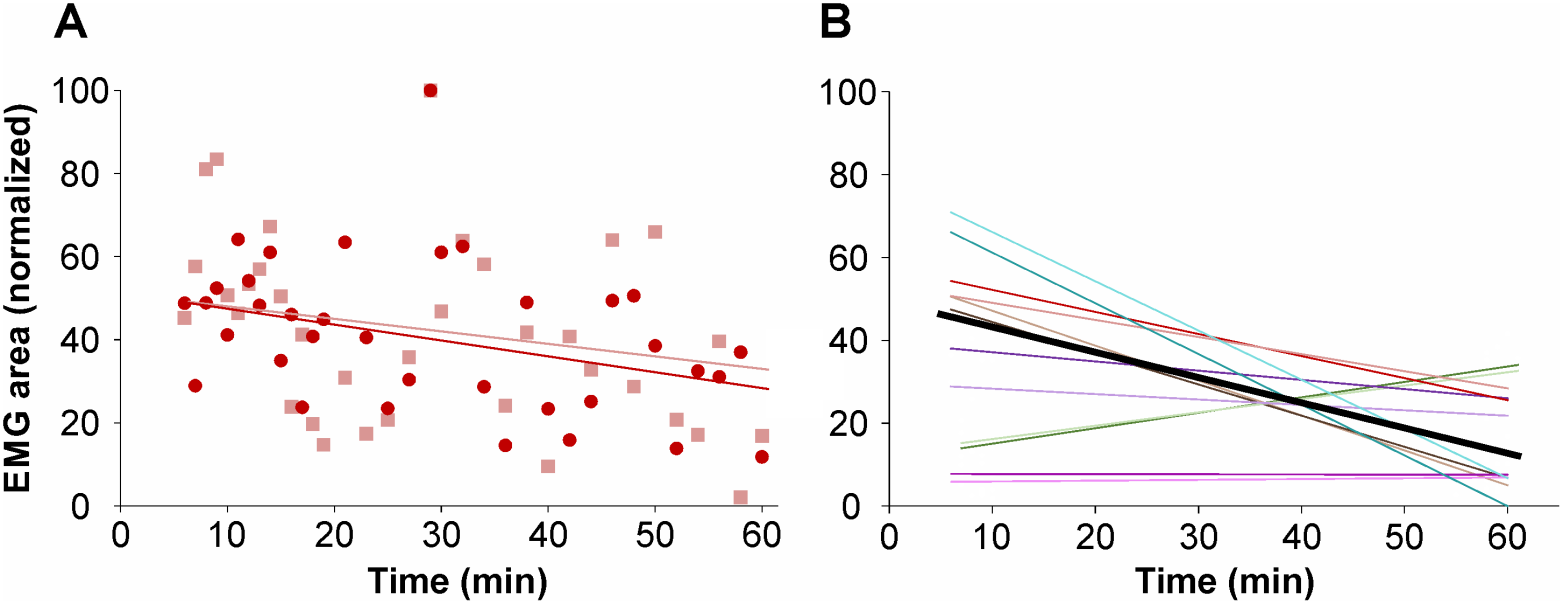
Triceps responses evoked by mechanical stimulation of the facial skin over time. (**A**) Responses (% EMG area, normalized) during 60 min. of stimulation in one specimen. (**B**) Correlation analysis for the 6 specimens tested. Each pair of colors, dark and light, represents the regression lines of the ipsi- and contralateral triceps of each given specimen and the black line is the average.

The effect of long term facial mechanical stimulation on triceps response was tested in 6 other preparations by applying a constant force of 6–7 g every 1 or 2 min during 60 min. Triceps response intensity decreased in 3 specimens (Fig 5A shows the results for one such specimen), remained at the same level in 2 specimens and showed a slight increase in 1 specimen (color lines in Fig 5B). Averaged, these results show a decrease from 34.2 ± 2.9% for the responses recorded after 10 to 20 min. of stimulation, to 19.9 ± 2.3% after 50 to 60 min. (*n* = 96 stimulations at each timeframe). In 4 of the specimens, weak triceps contractions could still be induced after 120 min. stimulation (data not shown).

Regardless of the strength or duration of the pressure applied to the snout, no rhythmical bouts of contractions, such as normally associated with locomotor rhythm, were induced, except once in 1 of all the specimens tested. Double or triple consecutive stimulations at different forces did not either induce rhythmical activity.

**Fig 6:**
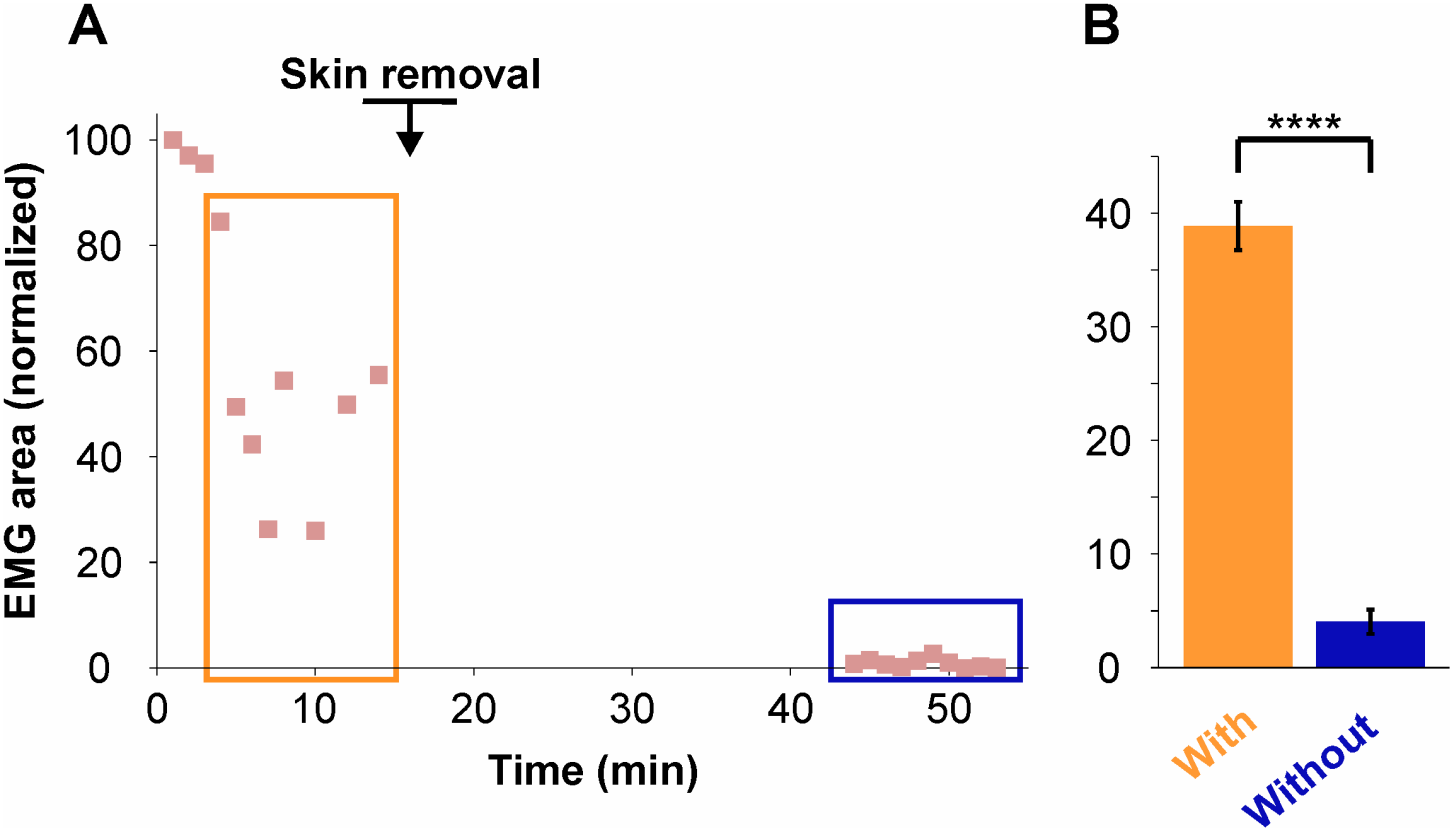
Triceps responses evoked by mechanical stimulation of the snout with and without the skin. (**A**) Responses (% EMG area, normalized) during a 10 min. interval in one specimen and during another 10 min. interval one half hour after snout skin removal, showing that the responses are nearly abolished. The orange and blue color boxes indicate the data from this specimen used in the histograms in B. (**B**) Histograms showing the average responses (% EMG area, normalized) ± SEM for the 5 preparations tested (*n* = 5).

To determine if the facial skin is necessary for the triceps to contract following pressure stimulation, we recorded the responses to mechanical stimulation in 5 additional preparations in which the snout skin was removed. After having first recorded the responses to stimulation with the skin on, the snout skin was gently cut out with microscissors, which took no more than 10 min. After a 30 min. rest, pressure of 6–7 g was applied to the skinless snout and the triceps EMGs were recorded. Fig 6A presents the results obtained in one representative specimen and shows that the responses were nearly abolished (blue box), compared to what they were before removing the snout skin (orange box). The average responses for the 5 specimens decreased from 38.88 ± 2.13% when the skin on (with; Fig 6B) to 4.03 ± 1.05% after skin removal (without; Fig 6B), this 90% reduction being highly significant (P < 0.0001, Mann-Whitney non-parametric t-test).

**Fig 7:**
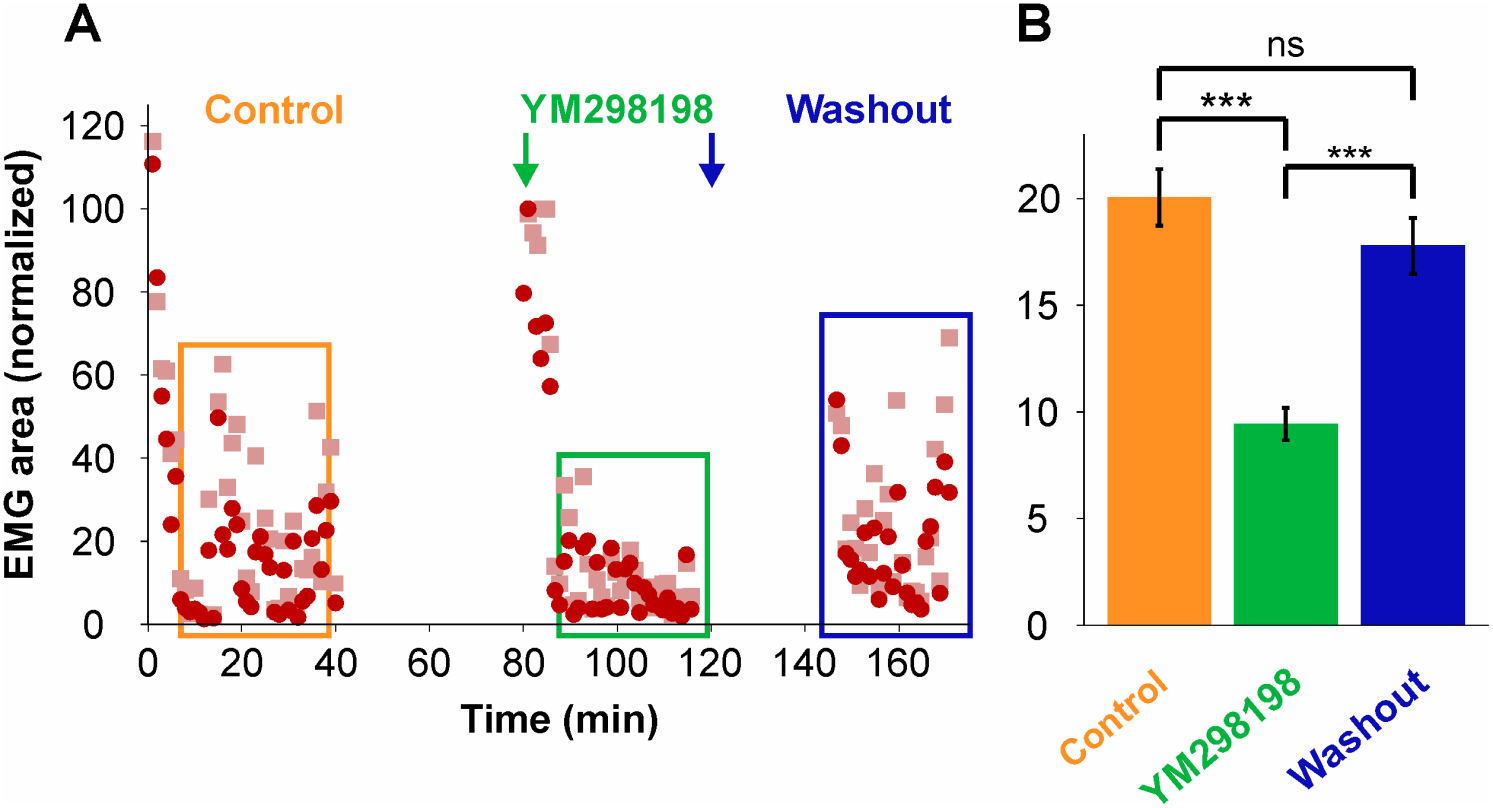
Effect of YM298198 bath application on triceps responses following mechanical pressure applied to the snout skin. (**A**) Responses (% EMG area, normalized) in one specimen before (orange box) and during (green box) YM298198 application, showing a decrease in the responses, and a reversal after washout (blue box). (**B**) Histograms of average responses (% EMG area, normalized) ± SEM for the 6 specimens tested.

To address the possible involvement of Merkel cells in the triceps responses induced by pressure applied to the skin, two pharmacological tests were carried out. Firstly, YM298198, the antagonist of the metabotropic glutamate receptor 1 involved in signal transmission between MC and the SA I trigeminal fibers that contact them [20], was applied to the bath ([] = 40 μM) in 6 preparations to test its effect on EMG intensity and latency. Fig 7A shows in one representative specimen that triceps EMG intensity decreased after YM298198 perfusion in the bath (green box) compared to before its application (control; orange box), and that the effect is reversible after rinsing the product (washout; blue box). The average response intensities for the 6 specimens tested (Fig 7B) were 20.1 ± 1.3% before YM298198 application, 9.42 ± 0.75%, during application and 17.8 ± 1.3% after rinsing (*n* = 236 stimulations in each condition). The decrease observed during YM298198 application is statistically highly significant (P < 0.0001, Kruskal-Wallis non-parametric one-way ANOVA with Dunn’s post-tests). YM298198 bath application also significantly increased the response latency of the triceps from 124.5 ± 15.5 ms to 216.3 ± 14.0 ms, an effect which is also reversible: 194.2 ± 15.0 ms after washout (result not shown; P < 0.0001; same ANOVA with post-tests).

**Fig 8:**
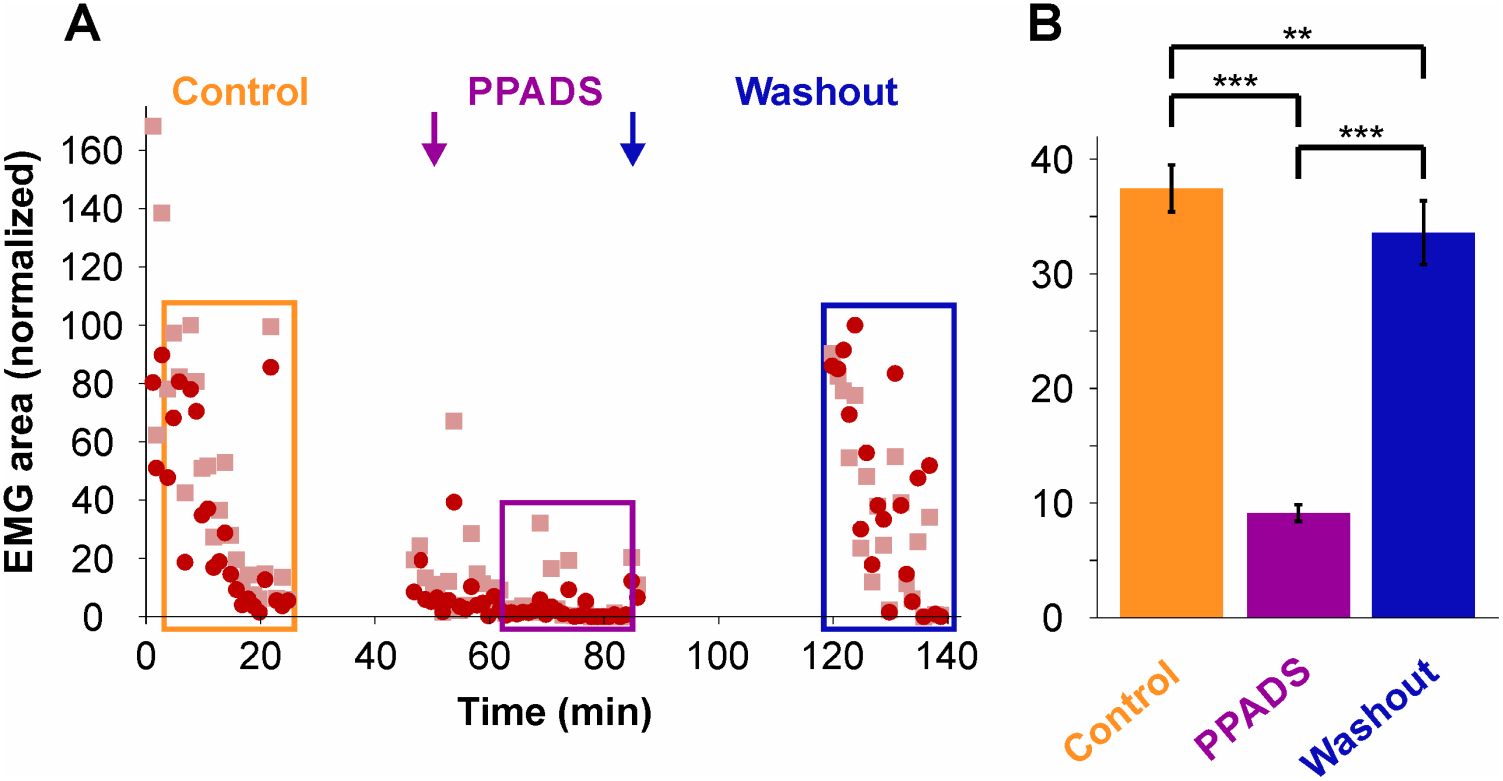
Effect of P2 antagonist, PPADS, on triceps response evoked by pressure applied on the snout skin. (**A**) Response (% EMG area, normalized) in one specimen before (orange box) and during (purple box) PPADS application, showing a decrease in the responses, and a reversal after washout (blue box). (**B**) Histograms of average responses for the 6 specimens tested.

Secondly, as will be discussed below, MC being in all likelihood the cells labeled by AM1-43 in the first set of experiments reported here, an antagonist to the channel used by the dye to enter cells was used to test its effects on triceps responses. PPADS, a P2 receptors antagonist which blocks AM1-43 entry into cells [17,19] and is associated with neuronal sensory endings [29–31], was applied to the bath in 6 specimens. As shown in Fig 8A for one representative specimen, bath application of PPADS (200 μM) significantly decreased the triceps EMG response intensity, and did so in a reversible manner. For the 6 specimens tested (Fig 8B), the responses averaged 37.5 ± 2.0% before PPADS application, 9.1 ± 0.7% during application and 33.6 ± 2.8% after washout (*n* = 169 stimulations in each condition). The 75% decrease observed during application of PPADS is statistically highly significant (P < 0.0001, Kruskal-Wallis non-parametric one-way ANOVA with Dunn’s post-tests) and suggests a disruption of P2 receptors in the mechanosensory networks. The application of PPADS to the bath also significantly and irreversibly increased the latency of the triceps responses, from 100.1 ± 11.0 ms to 247.7 ± 19.4 ms to 345.4 ± 22.2 ms (results not shown).

## Discussion

The present study aimed at determining if facial mechanosensation conveyed by the trigeminal nerve, more specifically via Merkel cells (MC), could influence motor activity of the forelimbs (FL) in newborn opossums and contribute to their guidance or attachment to the mother's nipples. Mechanical stimulations on one side of the snout skin readily induced bilateral contractions of the triceps, a FL extensor muscle, in *in vitro* preparations of the neuraxis with the limbs attached to the carcass. The two FL responses obtained were not alternate, as in normal locomotion, but rather synchronous, and were not rhythmical. The response amplitude was variable but increased with the force applied on the snout. Response intensity tended to decrease over time, which was expected and is probably explained by muscle fatigue, rather than adaptation to stimulation. Removal of the snout skin nearly abolished the EMG responses following mechanical stimulation, indicating that they resulted from stimulation of sensory afferents of the snout skin. The latter being devoid of Vater-Pacini or Meissner corpuscles in newborn opossums, we documented the presence of MC by using the AM1-43 tracer, revealing numerous labeled cells in the snout epidermis. To test for their functionality, an antagonist of the metabotropic glutamate receptors 1 (YM298198) was added to the bath and this halved the EMG response intensity following snout mechanical stimulation. Moreover, bath application of the antagonist of purinergic type 2 receptors PPADS, the receptor used by AM1-43 to enter sensory cells, decreased the response by 70%. These combined results suggest that mechanosensation in snout skin affects forelimb movements in newborn opossums and that this effect is mediated by MC innervated by the trigeminal nerve.

The FL motor responses being correlated to the pressure intensity exerted on the snout skin, this suggests that more mechanoreceptors were recruited as stimulus strength increased. This may also indicate an increase in their average firing rate. It cannot be excluded that other sensory modalities, such as nociceptors, were also recruited (see below). Whatever the strength of the pressure exerted on the snout, no locomotor rhythm was induced, except in one experiment in one specimen, and this episode may have been spontaneously generated. The absence of locomotor rhythm was also observed in our previous study following stimulation of the trigeminal ganglion to induce FL movement [10]. Comparable results were obtained for the rat hindlimbs following electrical stimulation of the trigeminal nerve and recording ventral roots and motoneurons in *in vitro* preparations of postnatal rats [32]. Yet, mechanical stimuli applied to the peripheral territory of trigeminal nerve innervation can induce locomotion in adult decerebrate cats, given prior injection of the GABAergic antagonist Picrotoxin in the pontomedullary locomotor region (which is contiguous to the ventral portion of the spinal trigeminal nucleus) [33–35]. Even if trigeminal stimulation does not induce locomotion, the present results using snout mechanical stimulation and FL EMG recording corroborate our previous report of FL movement following trigeminal ganglion stimulation and confirm without a doubt that facial mechanosensation affects FL movement in newborn opossums.

In the present study, muscular responses to a unilateral facial stimulation were, on average, of equivalent intensity and latency in both FL, just as in our previous study where the same threshold intensity of trigeminal ganglion stimulation elicited comparable FL movement on both sides [10]. Again, a comparable observation was made in postnatal rats [32], in which electrical stimulation of one trigeminal nerve generated comparable postsynaptic potentials in lumbar motoneurons and bursts of action potentials in the corresponding ventral roots bilaterally.

Given the bilateral and simultaneous triceps response to a unilateral snout stimulation, facial mechanosensation may not act as a guide for the newborn opossum to reach the teat, but as a tonic force to maintain the locomotor movement. When the opossum crawls on the mother’s belly, repetitive mechanical stimulation of the face via the trigeminal system may exert a tonic action on the spinal cord via brainstem or spinal relays which could sustain the locomotor activity. Once a nipple is reached, mechanosensation may trigger the animal's attachment to it [7]. Mechanosensation may act in conjunction with other senses, such as thermal or chemical, also conveyed by the trigeminal system. It is interesting to note that MC may also have chemosensory properties [13]. The thin and non-keratinized epidermis of the newborn opossum offers little barrier to the diffusion of volatile substances which could come from the mother's nipple. Even breathing can take place through the skin in newborn opossums [36].

Our experiments using the AM1-43 have shown numerous MC in the snout epidermis of newborn opossums. That nerve fibers were seldom labeled may be due to the short transport time (1 h) that was allowed here, compared to reports in older animals of other species [17,18]. As mentioned above, we already demonstrated the presence of numerous trigeminal fibers in the facial skin of newborn opossums using NF-200 as a marker [10].

The implication of Merkel cells in the observed triceps EMGs was corroborated by our physiopharmacological experiments using antagonists to glutamatergic and purinergic receptors. However, the substances having been applied to the bath, they may also have affected synapses other than those between MC and their innervating fibers (such as nociceptive) as well as central synapses. PPADS, for example, is known to block more or less efficiently P2X subtypes 1 to 5 (see [30]). The distribution of the different P2X and P2Y receptors has not been studied in opossums, but in rodents the presence of P2X2 and PX3 has been demonstrated in trigeminal and dorsal root ganglions [37–40]. These receptors are notably associated with putative nociceptive neurons (reviews in [30–31]). Investigating the distribution and expression of metabotropic glutamate 1 receptors and purinergic type 2 receptors in the brainstem and spinal cord of newborn opossums and assessing their functionality with more specific compounds would help resolve their possible role in early motor behaviors.

Considering that the nervous system of the newborn opossum generally compares to that of an E13 rat embryo (see [24,41]), the anatomical and physiological studies of prenatal rat trigeminal system development available [42,43] indicate that the rat lags the opossum by several days. The relatively more precocious development of this system in the opossum, as is also the case of its face, neck and forelimbs [44–46], may be explained by the requirements of the opossum's early birth and the need to reach a mother's nipple and attach to it. However, a recent, morphometric study of some components of the trigeminal system (vibrissae and their innervation, and trigeminal nuclei) in marsupials, monotremes and rodents has unveiled considerable differences between species in the pace of development relative to body size and indicates that the system may not be not more precocious in marsupials than in rodents [47].

In summary, the results obtained in this study support that mechanosensory inputs mediated by the trigeminal system, likely via Merkel cells, influence motor behaviors of the newborn opossum. The action on the forelimbs is tonic and likely serves as a reinforcement for the animal as it crawls on the mother's belly until it reaches a teat, where it may help its attachment to it.
